# Chitosan Composites in Packaging Industry—Current Trends and Future Challenges

**DOI:** 10.3390/polym12020417

**Published:** 2020-02-11

**Authors:** Victor G. L. Souza, João R. A. Pires, Carolina Rodrigues, Isabel M. Coelhoso, Ana Luísa Fernando

**Affiliations:** 1 MEtRICs, Departamento de Ciências e Tecnologia da Biomassa, Faculdade de Ciências e Tecnologia, Universidade Nova de Lisboa, Campus de Caparica, 2829-516 Caparica, Portugal; jr.pires@campus.fct.unl.pt (J.R.A.P.); cpe.rodrigues@campus.fct.unl.pt (C.R.); ala@fct.unl.pt (A.L.F.); 2 LAQV-REQUIMTE, Departamento de Química, Faculdade de Ciências e Tecnologia, Universidade Nova de Lisboa, Campus de Caparica, 2829-516 Caparica, Portugal

**Keywords:** chitosan, composites, food packaging, thermoplastic chitosan films

## Abstract

Chitosan-based composites play an important role in food packaging applications and can be used either as films or as edible coatings. Due to their high costs and lower performance (i.e., lower barrier against water vapor, thermal, and mechanical properties) when compared to the traditional petroleum-based plastics, the use of such biopolymers in large-scale is still limited. Several approaches of chitosan composites in the packaging industry are emerging to overcome some of the disadvantages of pristine polymers. Thus, this work intends to present the current trends and the future challenges towards production and application of chitosan composites in the food packaging industry.

## 1. Introduction

Chitosan films and coatings have been extensively studied in the past decades, since they are renewable, biocompatible, biodegradable, and non-toxic. Moreover, chitosan is a natural bio-active polymer with an inherent antimicrobial activity, which promoted its application, as a film or coating, for food preservation [1]. However, their performance, in terms of thermal, mechanical, and water barrier properties, needs to be improved, along with other technical barriers associated with its large-scale production at low cost [2], in order to replace the traditional environmental-unfriendly petroleum-based materials. Several approaches towards chitin/chitosan composite films and coatings in the packaging industry are emerging to improve the properties of the pristine polymers through incorporation of nanofillers and bioactive agents or as blends or bilayers with other biopolymers. Novel and more sustainable processes for chitin extraction and the biological production of chitin and chitosan are also being exploited as well as options for the scale-up of the process, through the use of novel plasticizers to obtain thermoplastic chitosan films. Therefore, this review intends to present the current trends and the future challenges towards chitin/chitosan composite films and coatings in the food packaging industry.

## 2. Application of Chitosan as a Coating

Coatings are defined as coherent layers formed from coating materials to a substrate, which can be either directly applied onto the surface of foods, as edible coatings, or onto the surface of packaging materials to functionalize them [3,4].

With respect of edible coatings, chitosan has been extensively studied to extend the shelf-life of food products, specially of fruits and vegetables (Table 1). Good reviews on this subject are available [5,6,7,8]. The coatings are applied and formed directly onto food product by addition of a liquid film-forming dispersion (with a paintbrush, fluidizing, spraying, or dipping) or of molten compounds [9]. In fruits and vegetables, coatings can retard ripening and water loss and reduce decay [10], while in meat products they can improve their quality by delaying moisture loss, enhancing product appearance, reducing lipid oxidation and discoloration, and also as carrier of food additives [11]. Moreover, chitosan coatings possess good oxygen and carbon dioxide barrier properties [12] and its intrinsic antimicrobial properties can also retard microorganisms development, synergistically extending the shelf life of the coated food [9,10,11].

Current trends in coatings are the incorporation of preservatives into the polymeric matrices, aiming to preserve the food and increase its shelf life. This type of coating (antimicrobial and/or antioxidant coatings) is an alternative to the conventional coatings for food, which protect only from water loss or against damage [13], and in the case of chitosan coatings, the active compound may enhance the intrinsic antimicrobial properties of this polysaccharide, thus, its preservative ability. Recently, the incorporation of active compounds of natural origin in biodegradable films or edible coatings is playing a significant role towards a more environmental friendly packaging [14]. The active compounds, such as natural extracts from plants, rich in phenolic compounds, or essential oils, are capable to enhance either antimicrobial and antioxidant properties of the chitosan, thus increasing the preservative properties of the coating and its ability to extend the shelf life of foodstuff [15,16,17]. In addition, this type of packaging is more attractive to the growing number of consumers looking for greener packaging options.

With the advancement of nanotechnology, new concepts, such as nanocoatings, which consist of ultra-thin nanoscale layers (less than 100 nm) built-up onto surfaces, are also being explored. This type of coating has the advantage of not modifying the surface topography of the material, while adding physical and chemical functions to the surface, such as altering gas barrier properties, surface hydrophobicity or conductive properties, to name a few [3,18].

Changing the surface of packaging materials can be done by several methods and techniques, which depend on the purpose of the material to be developed and can be divided in two groups: migratory or non-migratory active packaging. Examples of the former are through embedding, non-covalent immobilization or layer-by-layer deposition, and of the latter with photografting or covalent immobilization [3].

The process to make covalent grafting of active substances onto inert polymers starts with the functionalization of the polymeric substrate. Environmentally friendly methods (solvent-free), such as gamma-ionization radiation or cold plasma gas discharge, can be used to accomplish this step; subsequently the enriched surface with oxygen-containing groups is ready to bond with the active compounds, which can be added by dipping/immersion, spreading, or electrospinning [30,31,32,33].

The most efficient method, in respect to homogeneity of surface and thickness of deposited layer, to deposit chitosan into activated surface substrate of poly(lactic acid) (PLA) was by immersion, however electrospraying was the most versatile [33]. Moreover, coupling agents can also be used in the process (e.g., ethyl-3-[3-dimethylaminopropyl] carbodiimide hydrochloride or 1-carbonyldiimidazole, N-hydroxysuccinimide) [3,32,34]. Chitosan grafted onto a PLA surface enhanced the antibacterial and antifungal effect of the polymer, while also added antioxidant properties to the packaging material [33]. High molecular weight chitosan resulted in nanofibers in the polymer surface when electrospinning was used, which conferred to the polymer antioxidant activity and less material consumption, however, the immersion method resulted in stronger antimicrobial activity and more homogeneous surface. Coated PLA with chitosan also preserved the general aspect and properties of apple juice for a longer period of time (change in color or browning do not appear after 48 h storage) when compared to samples packaged in pristine PLA or in commercial plastic material (polyethylene terephthalate (PET)), in which the changes appeared after 24 h or 2 h, respectively [33]. Chitosan also enhanced antimicrobial properties of polyethylene (PE) when coated alone [34] or with vitamin E [32], demonstrating potential to be used either in food packaging or in medical applications.

## 3. Chitosan Films for Food Packaging

### 3.1. Blends and Bilayers of Chitosan and Other Biopolymers

Improvements in mechanical properties, better performance in terms of water vapor permeability and lower water solubility have been reported for combinations of chitosan with other polysaccharides, such as, starch, pectin, or alginate [35,36,37], microbial polysaccharides [38,39] and proteins, like gelatin [40] and whey proteins [41,42], compared to chitosan stand-alone films. This fact is attributed to the formation of polyelectrolyte complexes through electrostatic interactions between the protonated amino groups of chitosan and the negatively-charged side-chain groups in the other biopolymer at the operating pH [35,43]. Some authors reported difficulties in the total solubilization of one of the polymers in specific conditions and formation of insoluble complexes between polymers in blends preparation [41]. Bilayer systems can overcome this constraint, and reports show that these systems have better water vapor barrier properties than blend films [40,42].

### 3.2. Nanocomposites

The incorporation of nanoscale reinforcements (e.g., montmorillonite, nanocellulose, metal oxide nanoparticles) in chitosan films, that can interact chemically or physically with the polymeric chain, is an approach that seeks to rectify the intrinsic flaws, like low water resistance, poor mechanical and barrier properties, that are attributed to the hydrophilic nature of chitosan [17,44].

#### 3.2.1. Montmorillonite

Montmorillonite (MMT) is a layered silicate mineral clay, naturally present in volcanic rocks (bentonites), which is being pointed as a reinforcement material to bioplastics due to its wide availability, swelling and plasticizer ability, mechanical resistance, and low cost, just to mention a few characteristics [45].

Recently, several studies with nanocomposites based on chitosan and MMT have been investigated, and, in general, an enhancement in the mechanical and barrier properties is observed when MMT is incorporated in the chitosan film [16,17,46,47,48,49,50]. Beigzadeh Ghelejlu et al. (2016) [46], Giannakas et al., (2016) [51] and Nouri et al. (2018) [49], noticed that with low amounts of nanoclay in the bio-based films it is possible not only to improve strength, stiffness, and elongation at break but also increase water and oxygen barrier. In terms of optical properties, Souza et al., (2018) [45] showed that chitosan films with MMT exhibited remarkable light block barrier, especially at UV wavelength, acting as an extra protection against oxidation processes. Farther, was also demonstrated that these kind of nanocomposites have an increased antimicrobial activity [49,51]. Inspired by this Pires et al., (2018) [48] and Souza et al., (2018) [52] tested the nanocomposites in a perishable food matrix, demonstrating their potential to be used as primary packaging material, being capable of retarding deterioration process by antimicrobial and antioxidant mechanisms and extending its shelf life.

#### 3.2.2. Cellulosic Nanofibers and Nanocrystals

Cellulosic fibers in the nanoscale, namely cellulose nanofiber (CNFs) and cellulose nanocrystals (CNCs), are an appealing reinforcement in chitosan towards the production of environmentally friendly composite films with refined physical properties due to their highly compatibility with chitosan. The high interaction due to the electrostatic association and hydrogen bonds between nanocellulose with large length-diameter ratios and chitosan molecules causes the formation of an interactive network structure providing an increment in the films crystallinity [53,54]. Thereby, chitosan/nanocellulose composites have a large spectrum of applicability and potential in the field of biomedical, packaging and water treatment [55,56,57]. In two different studies, nanocrystalline cellulose was incorporated as reinforcing agent in chitosan-guar gum [58], starch-chitosan and gelatin-chitosan composites [59]. Both works accomplished a transparent and thermally stable biopolymer-based nanocomposite with improved mechanical and barrier properties. This new type of safe, non-toxic, renewable, and biodegradable chitosan/nanocellulose films, as a novel food packaging material, may one day replace petroleum-based polymers.

#### 3.2.3. Metal Oxides

Nanoscale metal oxides, such as ZnO, SiO_2_, TiO_2_, or MgO, add more value to chitosan due to their synergetic properties, including antimicrobial, UV blocking, and magnetic properties, in addition to their reinforcing ability [60,61,62,63]. Among the metal oxides, zinc oxide (ZnO) is one of the most broadly applied materials in several fields due to the notable antimicrobial and photocatalytic properties. ZnO nanoparticles in parallel with other metal oxide nanoparticles are regarded as safe materials for human beings, and have been used as food additives, packaging materials and in water purification [61]. In Youssef et al., (2015) [60] work, films loaded with ZnO nanoparticles showed antibacterial activity against *Staphylococcus aureus*, *Escherichia coli*, *S. typhimurium*, *Bacillus cereus*, and *Listeria monocytogenes*. Recently, Al-Naamani et al. (2016) [61] obtained successful results, showing that chitosan/ZnO coating on polyethylene films provided an effective antimicrobial defense against *S. enterica*, *E. coli*, and *S. aureus*, with fully-inhibited growth of the pathogens after 24 h incubation. Titanium dioxide (TiO_2_) is also an attractive inorganic nanomaterial which has lifted great interest in environmental and energy fields because of its low cost, high photocatalytic performance, high chemical stability, and biocompatibility [62]. The addition of TiO_2_ nanopowder has been reported to enhance the mechanical properties of the chitosan-based nanocomposite films [64,65]. The chitosan/TiO_2_ film produced by Zhang et al. (2017) [62] showed efficient antimicrobial activity against four tested strains, *Escherichia coli*, *Staphylococcus aureus*, *Candida albicans*, and *Aspergillus niger* with 100% sterilization in 12 h. Moreover, it induced the leakage of cellular substances through damaged membrane. Furthermore, the work of Silva et al., (2017) [66] highlighted that tensile strength and elastic modulus of chitosan nanocomposites with 5 (w/w%) MgO improved by 86% and 38%, respectively, compared to pristine chitosan. Chitosan nanocomposites with MgO nanoparticles also showed superior UV-shielding and moisture barrier properties. Therefore, the fabricated chitosan/metal oxides nanocomposite films with enhanced physicochemical properties can be used as a potential food packaging material.

### 3.3. Active Films of Chitosan

Microbiological growth and oxidative processes are two mechanisms responsible for food quality deterioration leading to important changes such as loss of nutritional values, texture modifications, development of undesirable compounds such as off-flavors, colored, and even toxic substances to humans [4]. Thus, active packaging plays an important role in the food industry, preventing wastes and promoting an increment in products shelf life [16]. In order to potentialize the innate characteristics of chitosan films, bioactive compounds like antimicrobial and antioxidants agents, gas scavengers, moisture absorbents and nutraceutical compounds, can be added. Due to the health concerns of the consumers, current research in active packaging has focused on developing natural preservative systems such as those based on nisin, lysozyme, essential oils, or fruit and plant extracts which exhibit antioxidant or antimicrobial properties which can be an alternative to those based on artificial additives and chemical preservatives [16,67,68]. However, the use of these natural compounds in food preservation is frequently limited because of their application costs and other disadvantages like their intense aroma and potential toxicity [69]. Thus, the design of an active package where there is no contact between the substance and the food constitutes an amazing opportunity with some advantages like no taste transfer, reduced organoleptic changes, and even distribution of the active compounds in the headspace [4].

Scientific research in the field of chitosan active packaging has been focused on the identification of the active biocompounds that confer better antioxidant and antimicrobial capacities to the edible films [15,68,70]. Moreover, further studies have been published to understand to what extent the introduction of these natural compounds affects the mechanical properties of the films [4,71]. Recently the films have been brought into contact with different food matrices in order to study their influence on the organoleptic properties of the food over the shelf life [16,17,72,73]. Lekjing (2016) [73] investigated the effects on quality and shelf life of cooked pork sausages coated with chitosan/clove oil, demonstrating that the combination of these two components inhibited microbial growth, retarded lipid oxidation, and extended the shelf life of cooked pork sausages for more than six days. However, there were some initially negative impacts on odor and taste attributes, at the start of storage time. In similar works, supplementation with ginger and rosemary essential oils also reduced the poultry meat oxidative processes [17], and Souza et al., (2019) [16] showed that in the in vitro essays, chitosan films with rosemary demonstrated good antimicrobial activity against *Bacillus cereus* (reduction of 7.2 log) and *Salmonella enterica* (reduction of 5.3 log). Briefly, the bioactive agents incorporated in chitosan films showed great promise for their application in extending the shelf-life and maintaining the quality of food products and controlling postharvest fungi and foodborne bacteria in food system. Extra work needs to be done to understand the interactions between chitosan and bioactive compounds in order to optimize the effectiveness of the bioactive incorporated agents. Moreover, most of the studies use the casting method for the production of the polymers, which is a technique not readily applicable by the packaging industry, unlike compression molding or extrusion, in which the material is submitted to high temperatures. Thus, new tests and studies should be conducted to overcome the challenge on how to keep the antimicrobial/antioxidant activity of essential oil/extracts in the films during the high temperature of the plastic production processes [74].

Adding a wide range of lipid components, natural waxes, resins, fatty acids and vegetables oils, to films will also confer hydrophobicity to the film and reduce moisture [75]. A decline in water solubility has been reported for chitosan films with beeswax [76] and a decrease in water vapor permeability was described for films with oleic acid [77], neem-oil [78], and cinnamon essential oil [79], among others.

**Figure 1:**
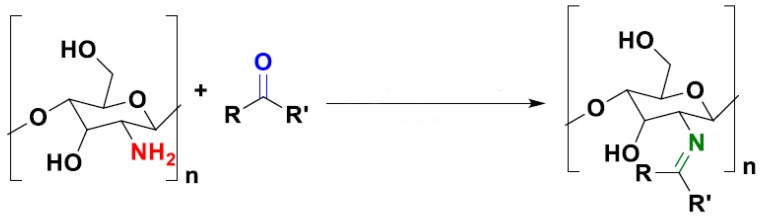
Chemical modification of chitosan via imine functionalization.

The intrinsic reactive groups of chitosan, namely, OH and –NH_2_, allow the chemical modification of chitosan, enhancing its application potential. The reaction between chitosan’s amino groups and carbonyl compounds via imine functionalization results in chitosan-based Schiff bases, which are of importance for certain food packaging applications (Figure 1). Chitosan-based Schiff bases have shown antimicrobial activities as powders/whiskers/films/membranes, and, interestingly, exhibiting better antimicrobial properties than bare chitosan [80]. Moreover, the antimicrobial action of chitosan-based Schiff bases can be augmented by loading metal ions or metal nanoparticles through the covalent coordination bond. Some chitosan-based Schiff bases have also shown antioxidant activities improving this way the functional properties of bare chitosan. Some examples are the Schiff bases obtained from the reaction of chitosan with D-fructose, quercetin o-quinones, eugenol aldehyde, or carvacrol aldehyde [80].

## 4. Novel Extraction Methods of Chitin and the Production of Chitosan

The main source of commercial chitosan is chitin, which is the second most abundant polysaccharide on Earth, only preceded by cellulose. It is present in green algae, the cell walls of fungi, the cuticles of insects and arachnids, and in the exoskeleton of crustaceans. At the industrial scale, the main source of chitin are the shells from crustacean (shrimp, prawn, crab, and lobster) processing industries. The main components of crustacean shells are chitin (15%–40%), protein (20%–40%), calcium and magnesium carbonate (20%–50%), together with other minor constituents, such as astaxanthin, lipids, and other minerals [81,82].

**Figure 2:**
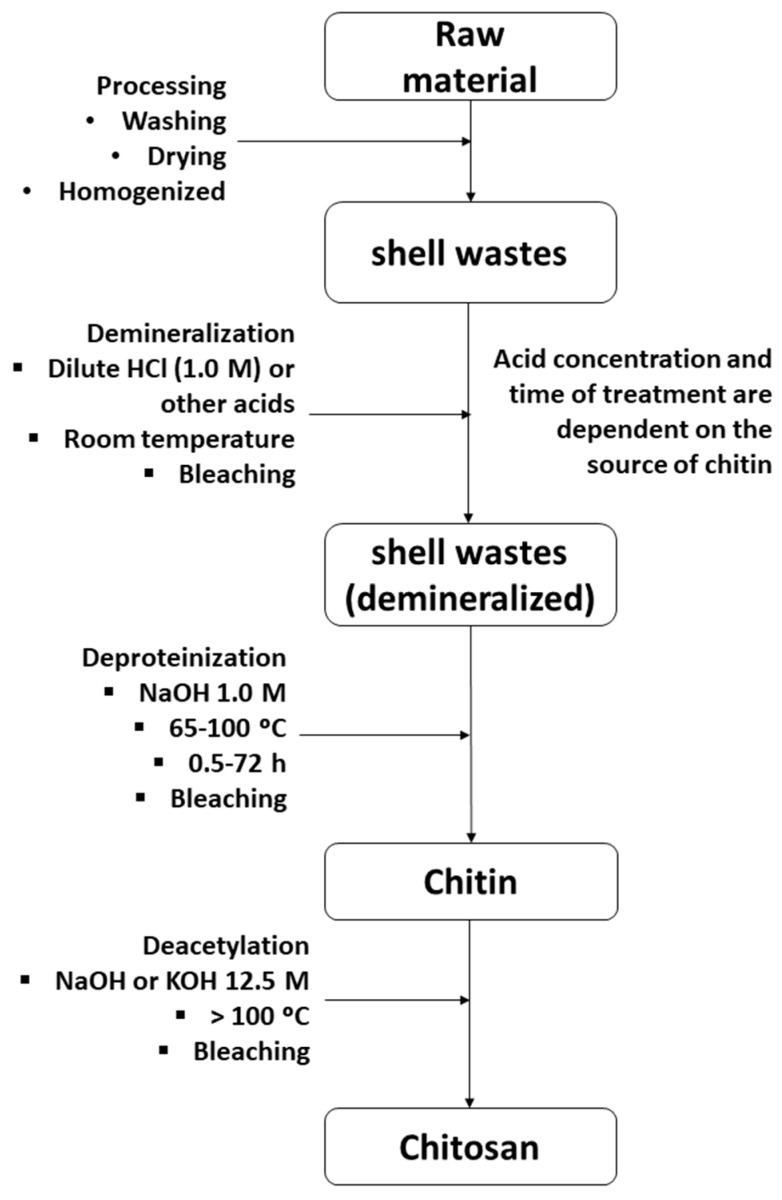
Conventional chemical production process of chitosan from shells of crustaceans.

Chitosan production involves several chemical processes, such as decalcification, deproteinization, decolorization and deacetylation (Figure 2) [83]. Demineralization of the shells is usually carried out with dilute HCl solutions at room temperature, although other acids may also be used (HNO_3_, H_2_SO_4_, CH_3_COOH). The acid concentration and the time of treatment depend on the source of chitin. Deproteinization of the shells is performed with dilute NaOH solutions at 65–100 °C for 0.5–72 h. Deacetylation of chitin to produce chitosan is usually achieved by hydrolysis of the acetamide groups with concentrated NaOH or KOH (40%–50%) at temperatures above 100 °C. This reaction is generally carried out under heterogeneous conditions. The acetylation degree (DA) of chitosan, defined as the proportion of acetylglucosamine units in the polymer, will depend on the deacetylation conditions. It is very difficult to completely deacetylate chitin without using specific procedures, so that the DA of chitosan generally lies between 40% and 13%, while its molecular weight ranges from 2 × 10^5^ to 1 × 10^6^ Da.

To avoid acidic and alkali treatments, which are extremely hazardous to the environment, biological treatments are an alternative method to extract chitin and produce chitosan. Lactic acid-producing bacteria and proteases from bacteria have been used on the demineralization and deproteinization steps, respectively. Chitin deacetylation is carried out using enzymatic methods by chitin deacetylase. Despite the high quality of final product and being a method environmentally safe it takes a long processing time (several days) and is until now limited to laboratory scale studies [84].

In general, chitosan is prepared using conventional chemical and enzymatic methods, but ultrasound technology is added in some cases to improve the chitosan proprieties. Several studies on the impact of ultrasound on chitosan molecular weight and deacetylation degree have been published confirming that ultrasound-assisted extraction of chitosan significantly reduces its molecular weight [85,86,87].

Microwave technology can also be used as an excellent alternative to conventional thermal heating because it offers increased reaction rates, shorter reaction times, higher yields, energy savings, and a reduction of side reactions. Moreover, microwave technology can be very effective for chitosan depolymerization and is a promising method for obtaining low molecular weight chitosan. This ecological method dramatically reduces the extraction time of chitosan to few minutes compared to the conventional method that requires several hours. Thus, microwave technology can be a rentable method to scale-up chitosan production [88].

A novel approach in the extraction of chitosan is the use of deep eutectic solvents (DES), a new class of green solvents. DES are obtained via simple and convenient methods, are non-toxic or have low toxicity, are biodegradable, and the large number of composition possibilities allow tailoring their properties and applications. DES are also highlighted as possible alternatives for ionic liquid solvents [89].

DES were successfully applied for dissolving chitin, avoiding alkali treatment for dissolution or transformation into chitosan via deacetylation [89,90]. Sharma et al. (2013) tested various DES types: choline bromide/urea, Choline Chloride (CC)/thiourea, choline chloride/urea, and betaine hydrochloride as α-chitin (from crab shells) solvents. The highest chitin dissolving efficiency was 9 wt% in a mixture of CC and thiourea (1:2 M ratio) heated for 6 h at 100 °C (for CC/urea: 6 wt% heating for 10 h at 100 °C). Applying microwave and ultrasonication led to time and temperature reductions necessary for chitin dissolution. Moreover, chitin derivative-chitosan was not dissolved in CC/thiourea, indicating interactions between O=CNH groups (from glucosamine unit), and thiourea might play a role in the dissolution process. Zhu et al. (2017) [91] tested CC-based DES (with glycerol, urea, thiourea, or malonic acid) as an extracting media for chitin from lobster shells. Using CC/malonic acid (1:2 M ratio), allowed to isolate polysaccharide with high purity, effectively removing proteins and minerals and a yield of 21% higher than the chemically prepared chitin, which is 16.5%. Moreover, chitin isolated and purified with CC/malonic acid is characterized by giving two materials with different crystallinity and thermal stability. Ramírez-Wong et al. (2016) [92] showed that treatment of chitin (after acidic purification) with CC/U caused crystalline phase transition (γ-mono-phase) and increase thermal stability after precipitation/water evaporation.

## 5. Biological Production of Chitin and Chitosan

The production of chitin and chitosan-derived products is currently dominated by chitin obtained from waste shellfish, which may limit its scale-up, due to volatile resource availability, to the presence of residual allergens and contaminants which require costly purification and refining of the final product, and to the low degree of deacetylation [93]. The biological production of chitin and chitosan is being considered an alternative process to ensure the availability and to reduce the allergen problematic issue. Algae are a source of chitin and chitosan, along with other added value compounds, such as lipids. The centric diatoms *Cyclotella* sp. and *Thalassiosira* sp. produce extracellular nanofibers composed of β-chitin, that can be extruded from the cell [94]. The controlled cultivation of these diatoms offers a source of this nanomaterial [95]. Chitosan can also be produced by fungi. Zygomycetes are microorganisms capable of producing fungal chitosan, where chitin is synthesized by chitin synthase and stored in the cell wall, being transformed into chitosan by chitin deacetylase [96]. The fungal biomass can be obtained by simple fermentation at very low cost and its extraction is considered a green process once it does not require a demineralization step [97]. Additionally, fungal chitosan has a medium-low molecular weight, by comparison with the one extracted from crustaceans, with a higher bioactivity [98]. The quality and quantity of chitosan extracted from fungi (including mushrooms and mushroom wastes), depend on the microorganism, type of fermentation, culture medium composition, pH, temperature, and extraction time and process [98,99]. The use of synthetic media, which is costly, can be substituted by low-cost carbon sources, such as agro-industrial wastes, promoting the bioeconomy and circular economy and reducing the environmental impact by avoiding its disposal into the environment [100]. Industries using fungi in different processes, such as brewing and baking, antibiotics and pharmaceuticals, produce thousands of tons of waste fungal biomass every year. Extraction of high levels of chitosan from these industrial fungi is also an alternative [101]. Current research is also focusing to increase the content of fungal chitosan in the cell walls, through strain improvement and metabolic engineering [101].

## 6. Scale-Up Production

The manufacturers and suppliers of chitosan and chitin products are present worldwide. Primex (Iceland) commercializes ChitoClear^®^, chitosan products that pretend to be based on the purest chitosan possible with potential application in food packaging. Norwegian Chitosan (Norway) trades chitin and chitosan under brand names NorLife and Kitoflok™, respectively, for several applications, including food and beverages. G.T.C. Bio Corporation (China), which is a chitin and chitosan manufacturer, commercializes different grades of both products with a price around 20 €/Kg for chitin and between 18–45 €/Kg for chitosan (depending on the required purity grade) [102].

Chitosan films and coatings have been extensively studied in the past decades, however, most of the data available consist in the production using the casting methods, mainly used in laboratory scale. The casting method consists of dissolving the polysaccharide in a suitable solvent, which for chitosan is generally an acetic acid solution, and simultaneously incorporate the active compound, the plasticizer and the nanofiller of interest, followed by pouring the resulting mixed dispersion onto an inert surface to evaporate the solvent and obtain the thin film. Thus, one challenge in the use of chitosan is the translation of this laboratory-scale method to an industrial one, or to find alternative production methods that could substitute the casting methodology.

Adding plasticizers (e.g., glycerol) to chitosan films, and applying thermomechanical treatment (mechanical kneading), allows to obtain a kind of thermoplastic material which grants good mechanical properties [103]. This process of plasticization by thermomechanical treatments can be a potential alternative to the traditional casting method of chitosan film production, which may also allow the preparation of these biodegradable films on a larger scale [104].

### 6.1. Thermoplastic Chitosan Films

The thermomechanical kneading approach was used to test different plasticizers on the production of chitosan film [105]. Different non-volatile polyol plasticizers (glycerol, xylitol, and sorbitol) were studied, the thermomechanical treatment was done in an internal mixer in the presence of water, acetic acid, and polyol investigated. Sorbitol (the highest molecular weight polyol tested) resulted in plasticized chitosan with the highest thermal, mechanical, and rheological properties, while the films produced with glycerol (the lowest molecular weight polyol) had the lowest thermal, mechanical and rheological properties, but the highest amorphous phase content, which made its processability easier, despite its poorer properties [105].

More recently, chitosan was plasticized using a one-step extrusion process in the presence of glycerol and acetic acid solution, and mixed with polyethylene to produce blends with different content of plasticized chitosan [106]. The resulting films presented a brown color and increasing haze with chitosan plasticized content, and the mechanical and oxygen barrier properties of the polyethylene films were nearly unaffected by the presence of plasticized chitosan, while the water vapor permeability increased with the amount of the incorporated carbohydrate [106].

Similar results are reported for biodegradable blends of thermoplastic starch with plasticized chitosan obtained by thermocompression [107], blown extrusion [108,109], and melt extrusion [110]. In this regard, the extrusion processes allow the preparation of plasticized chitosan-based materials on an industrial scale, which may overcome the scale-up problem of producing chitosan films.

### 6.2. Novel Green Solvents as Plasticizers for Thermomechanical Treatment of Chitosan—Deep Eutetic Solvents (DES)

Chitosan can be also processed using DES. Galvis-Sánchez et al. (2016) [111] prepared thermocompression molded films with chitosan (deacetylation degree 90%), CC, and citric acid (CA) (molar ratio 1:1). CC and CA were added separately to chitosan (not as a liquid mixture), and this three components system was heated for 30 min at 70 °C and then 3% acetic acid solution was added, and the formed paste was hot-pressed at 120 °C. In comparison to chitosan/CA films, chitosan/CC/CA ones exhibited higher water sorption ability. Moreover, incorporation of CC into chitosan/CA matrix resulted in tensile strength decrease, and slight elongation at break increase. Similar results were obtained by Almeida et al., (2018) [112] where CC/lactic acid was used as a plasticizer for chitosan films with curcumin. Chitosan/microcrystalline cellulose films (plasticized with CC/G in the presence of curcumin) can be applied as pH-responsive materials [113].

Natural deep eutectic solvents (NADES) prepared from cheap raw materials were tested to produce thermoplastic chitosan films [104]. Four types of NADESs based on choline chloride prepared with malic acid (MA), lactic acid (LA), citric acid, and glycerol were used as hydrogen bond donors, and as polymeric matrix, two chitosan with different deacetylation degrees (DD) (DD = 76 and 81). Transparent thin chitosan films were produced by thermo-compression molding, and film properties (mechanical and water resistance) varied depending on its composition/structure. A more homogenous surface, compact with lower water permeability and stronger resistance, were obtained for chitosan with lower DD and with the NADES CC/CA and CC/MA, while films produced with CC/glycerol resulted in a material with weaker properties [104].

Therefore, DES and NADES are suitable green solvent materials to be used as plasticizers to produce chitosan thermo-compressed films with tailored properties at large scale.

## 7. Conclusions and Final Remarks

Chitosan films and coatings have been extensively studied in past decades for food preservation, since they are biocompatible, biodegradable, and bioactive. However, their performance, in terms of thermal, mechanical, and water barrier properties, needs to be improved in order to be produced in large scale at low cost. Blends and bilayers with other biopolymers, as well as nanocomposites, have been developed to improve the mechanical and barrier properties.

Microwave technology can be a rentable method to scale-up chitosan production, since it reduces from hours to minutes the extraction time of chitin from waste shellfish. To avoid acidic and alkali treatments, which are extremely hazardous to the environment, biological treatments are alternative methods. Another novel approach in the production of chitosan is the use of deep eutectic solvents (DES), a new class of green solvents, which can be obtained via simple and convenient methods.

Options for improvement also rely on the biological production of chitin and chitosan, which will minimize resource availability and allergen-problematic issues, however, low-cost carbon sources, such as agroindustrial wastes, should be used, promoting a circular economy and reducing the environmental impact. Plasticized chitosan films using green plasticizers (DES and NADES) that can be obtained by extrusion processes is also a promising technology that allows their production at an industrial scale.

## Figures and Tables

**Table 1:** Chitosan edible coatings studies.

Food Applied	Active Compound Incorporated	Storage Condition	Key Findings	Ref.
Mango (*Tommy Atkins*)	-	23 °C	The coating delayed the ripening of semi-ripe mangoes stored at 23 °C. The level 1.5% provided better maintenance of physical chemical parameters assessed.	[19]
Sliced Mango	-	6 °C	Chitosan coating retarded water loss and the drop in sensory quality, increasing the soluble solid content, titratable acidity and ascorbic acid content, and inhibiting the growth of microorganisms.	[20]
Chicken breast	0.25% oregano essential oil (OEO)	4 °C	The shelf-life of chicken fillets was extended using, either OEO singly, and/or chitosan, by approximately 6 (with only OEO) and more than 15 (chitosan coating with or without OEO) days. Treated chicken samples with the coatings were sensorially acceptable during the entire refrigerated storage period (21 days), not negatively influencing the taste of chicken samples	[21]
Egg	-	25 °C	The coating created a protective barrier against the transfer of moisture and carbon dioxide through the eggshell, keeping a high Haugh unit and yolk index, while preserving it from *Salmonella enteritidis*.	[22]
Fresh fillets of Atlantic cod (*Gadus morhua*) and herring (*Clupea harengus*)	-	4 °C	Potential of chitosan as a preservative coating in reducing or preventing moisture loss, lipid oxidation, and microbial growth	[23]
Guava (*Psidium guajava* L.) fruit	-	11 °C	Fruits coated with 2.0% chitosan reduced weight and firmness loss, delayed changes in chlorophyll and malondialdehyde contents and soluble solids content, retarded the loss of vitamin C and the decrease of titratable acidity, during 12 days of storage, delaying ripening process.	[24]
Blueberry (*Vaccinium corymbosum*) fruit	*Aloe vera* extract	5 °C	Microbiological growth and water loss levels were approximately reduced by 50% and 42%, respectively, in coated blueberries after 25 d compared to uncoated blueberries. The chitosan coatings with the extract have proven to have great potential in expanding the shelf- life of fruits.	[25]
Fresh cut broccoli	Bioactive compounds and essential oils	5–7 °C	Pristine chitosan coating or enriched with bioactive compounds/essential oils resulted in a significant reduction in mesophilic and psychotropic counts. The enrichment with active compounds improved the antimicrobial action of chitosan. The application of these coatings did not introduce deleterious effects on the sensory attributes of broccoli.	[26]
Walnut kernels	Tea extract	Room temperature	Effective inhibition of lipid oxidation and fungal growth during storage of walnut kernels (18 weeks) with chitosan coating combined with green tea extract. No significant effect on sensory properties was observed during storage time. The results suggested that the active coating could prolong the shelf life of walnut kernels	[27]
Fresh-cut pears	Rosemary extracts	20 °C	The study suggests that chitosan + rosemary extract coating have the potential to improve the quality of fresh-cut pears and extend the shelf-life, by reducing changes in pH, inhibiting polyphenol oxidase activity, weight loss, and retaining high firmness and soluble solid content.	[28]
Guava (*Psidium guajava* L.) fruit	Pomegranate peel extract	10 °C	Samples coated with chitosan enriched with bioactive extract proved to be an effective treatment to maintain the overall fruit quality during 20 days at low temperature storage.	[29]
